# IMRMB-Net: A lightweight student behavior recognition model for complex classroom scenarios

**DOI:** 10.1371/journal.pone.0318817

**Published:** 2025-03-10

**Authors:** Caihong Feng, Zheng Luo, Deyao Kong, Yunhong Ding, Jingyu Liu

**Affiliations:** 1 Department of Computer Science and Information Engineering, Harbin Normal University, Harbin, China; International University of Languages and Media: Libera Universita di Lingue e Comunicazione, ITALY

## Abstract

With the continuous advancement of education informatization, classroom behavior analysis has become an important tool to improve teaching quality and student learning outcomes. However, student classroom behavior recognition methods still face challenges such as occlusion, small objects, and environmental interference, resulting in low recognition accuracy and lightweight performance. To address the above problems, this study proposes a lightweight student behavior recognition model based on Inverted Residual Mobile Block (IMRMB-Net). Specifically, this study designs a lightweight feature extraction module, IMRMB, from the images of the backbone network to be able to better capture contextual information and improve the recognition of occluded objects while saving computational resources. Using DySample, the neck network reconsiders the initial sampling position and the moving range of the offset from the point sampling perspective to accurately recognize small object behaviors in course scenes. Meanwhile, a new loss function, Focaler-ShapeIoU, is designed in this study, aiming to improve the learning ability and robustness of the model to different samples thus further solving the occlusion problem. Experiments in UK_Dataset show that IMRMB-Net has high accuracy (mAP@50 = 93.3%, mAP@50:95 = 78.7%) and lightweight performance (FPS = 60.37, Params = 7.32MB, GFLOPs = 23.8G). Meanwhile, this study verifies that IMRMB-Net can effectively solve the occlusion problem in classroom scenarios through experiments on the UK_Dataset and SCB_Dataset occlusion subsets. In addition, this study verifies the generalization ability and the ability to recognize small targets of IMRMB-Net on the VisDrone2021 dataset.

## 1. Introduction

Recognition of students’ classroom behavior is an important area of research in the field of education. By recognizing and analyzing students’ behaviors in the classroom, teachers can gain a better understanding of the student’s learning status, which can lead to improvements in the quality and effectiveness of instruction [1]. Traditional methods of analyzing classroom behavior primarily rely on manual observation and recording, which is labor-intensive, time-consuming, and subject to subjectivity[2]. As a result, the use of computer-assisted teaching to automatically recognize and analyze students’ classroom behavior has shown up as a hotspot in the field of intelligent education research [3,4].

Human posture estimation, which identifies key points on the human body to estimate posture and subsequently analyze student behaviors, has been used by a number of studies to deploy student behavior recognition in classrooms [5]. For instance, skeleton pose estimation and person detection are the foundations of the student behavior recognition system that W Zejie et al. suggested [6]. However, occlusion and other elements that impact the assessment of human postures make it challenging for this method to reliably identify student behaviors in complex classroom conditions [7]. Actually, object detection-based algorithms have advanced significantly in the last few years [8]. Therefore, in order to recognize student behavior in the classroom, an algorithm based on object detection is utilized in this research.

Prior to the development of deep learning, traditional object detection algorithms relied on manually created features, such as those for Sobel edge detection, Haar, Hog, and other features. These features have limited generalization capacity and exhibit subpar performance in intricate scenarios [9]. Convolutional neural networks (CNNs) are used by deep learning-based object detection algorithms to learn features. This feature-learning technique is capable of recognizing the features required to detect and classify the object while also transforming the original input data into more abstract, higher-dimensional features through the network. These high-dimensional features have strong feature expression and generalization capabilities, so their performance is superior in complex scenarios [10].

Object detection still faces a number of difficulties, though, when it comes to identifying classroom behaviors [11]. In actual classroom scenarios, occlusion frequently happens between students as well as between students and desks and chairs. This causes visual elements to become fragmented and reduces the precision of behavior recognition. Furthermore, pupils seated toward the back of the class have fewer visual elements captured by the camera, which may result in missed or incorrect detection. The number of samples for various behavioral categories in classroom behavior recognition may be unbalanced, and extrinsic distractions such as the surrounding environment, angle, and other individuals may influence students’ behavior. In summary, the aforementioned difficulties result in a notable decline in the precision of recognizing behavior among students. Furthermore, the computing power of classroom cameras is typically restricted, necessitating the deployment of object identification models with a small number of parameters that yet achieve high recognition accuracy.

**Fig 1:**
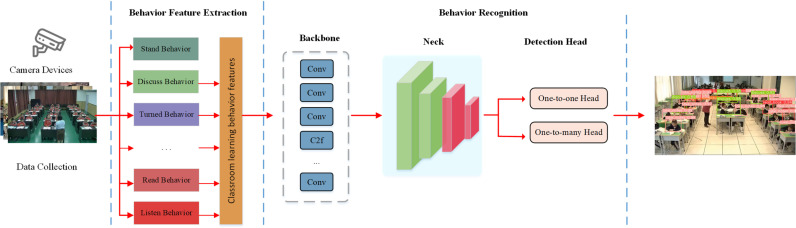
The workflow of student classroom behavior recognition.

This study aims to enhance recognition accuracy while increasing processing speed and minimizing computational resources. To meet the aforementioned issues, it presents an effective and lightweight recognition network, called IMRMB-Net, to recognize eight classroom behaviors performed by students. Fig 1 displays the framework’s workflow. Continuous video frames from the classroom camera are fed into the framework at first. The three primary stages of the process are the gathering of data on student behavior in the classroom, feature extraction, behavior recognition, and recognition.

Since there is severe occlusion among students in the classroom scenarios, as the input image illustrates, we designed an efficient lightweight attention mechanism, IMRMB, to combine two attention mechanisms, iRMB and MLCA, to optimize the feature extraction and recognition process. This increases recognition accuracy and lowers computational resources by capturing contextual information while accounting for channel information and spatial information consumption. Furthermore, since students located in the back corners of the classroom have smaller image sizes and distorted visual features[12], we employ the DySample structure in the neck network, which allows the network to focus on the object area more flexibly by dynamically adjusting the sampling position, particularly with better responsiveness to small objects. Finally, to address the challenges posed by lighting, angle, and occlusion on behavior recognition, as well as sample imbalance in actual classroom scenarios [13], this study combines the Focaler-IoU loss function with the Shape-IoU loss function to form the Focaler-ShapeIoU loss function. This combined loss function focuses on samples that are challenging to classify while maximizing shape similarity. This improves the model’s performance in the face of these disruptive factors. To summarize, this research work’s primary contributions are as follows:

1. This study constructed a dataset(UK_Dataset) of students’ classroom behaviors in a real classroom and classified students’ behaviors into eight categories.
2. In this study, we designed an effective lightweight attention mechanism IMRMB, which can help the detection model to better capture contextual information, improve the recognition of occluded objects, and increase the processing speed while improving the performance of the object detection model.
3. In this study, the DySample structure is used in the neck network to improve the recognition accuracy of small objects and make the model more effective in recognizing dense scenes.
4. In this study, Focaler-ShapeIoU is designed as a loss function for recognition. Combining the advantages of Focaler-IoU and Shape-IoU solves the problem of unbalanced training samples and makes the model more robust in the face of disturbing factors such as light and occlusion.
5. In this study, an efficient and lightweight student behavior recognition network (IMRMB-Net) is proposed, which can effectively solve the problems of occlusion, small objects, and interference. In this study, the IMRMB-Net detection network framework is tested on UK_Dataset, and the detection performance is improved while reducing computational resources. And the ability of IMRMB-Net to solve the occlusion problem in classroom scenes was verified on the occlusion subset of UK_Dataset and SCB_Dataset. Finally, the ability of IMRMB-Net to recognize small objects in everyday scenes and its generalization ability were evaluated on another dataset.

## 2. Related work

### 2.1 Object detection

In the discipline of computer vision, object detection is a crucial subject that seeks to identify the locations and classifications of every object in a picture or video [14]. object detection algorithms have advanced significantly in recent years because of the quick development of deep learning techniques, particularly the use of convolutional neural networks (CNN) [15]. Two-stage detectors and single-stage detectors are the two primary categories into which deep learning-based object detection techniques fall. Two-stage detectors such as Fast R-CNN, Faster R-CNN, and Mask R-CNN. These techniques initially produce candidate regions, on each of which they do bounding box regression and classification. By using the region proposal network (RPN), the Faster R-CNN greatly increases the candidate region generation’s speed and quality [16]. Particularly in complicated scenarios, the detection accuracy is excellent, but the detection speed is slow and the computational resource consumption is considerable. Single-stage detectors, the YOLO family and its variants such as YOLOv1 [17], YOLOv2 [18], YOLOv3 [19], YOLOv4 [20] and YOLOv5 [21]. In order to forecast the object’s location and class, these techniques employ direct global regression on the image [22].SSD techniques can handle objects of various sizes and can be detected at several scales [23]. The single-stage detector can be used in real-time applications because of its quick detection speed. But when it comes to small objects and complicated situations, the detection accuracy is, marginally worse than with two-stage approaches.

### 2.2 Traditional classroom behavior recognition methods

Meeting the demand for large-scale, real-time behavior recognition in the classroom is challenging due to the subjectivity and inefficiency of human-dependent behavior recognition [24]. Using machine learning techniques, several researchers have attempted to apply the recognition of student behavior in the classroom. The following procedures are typically involved in these methods: hand-designed methods for feature extraction from video data, such as color histograms, edge features, shape features, etc. Algorithms are used to filter the most representative features and eliminate duplicate data. Classification is done using machine learning methods like Decision Tree, K Nearest Neighbor (KNN), Support Vector Machine (SVM), etc. To identify students’ classroom behaviors in real-time, Chonggao Pang, for instance, combined the traditional cluster analysis algorithm and random forest algorithm with the human skeleton model. The results of the algorithm performance test demonstrated that the network structure of the proposed algorithm was superior to the single-feature extraction algorithm [13]. By combining the PSO and KNN algorithms, Shilong Wu created the PSO-KNN joint algorithm. This method, along with the emotional image processing technique, allowed Wu to build an artificial intelligence-based model for recognizing student behavior in the classroom. The findings demonstrate that the combined algorithm has a high accuracy rate in identifying pupils’ emotions and behaviors [25]. Xuyun Zhang examined the aberrant behaviors in the classroom using the Gaussian high-dimensional random matrix approach. They created a behavioral dictionary using the Orthogonal Gaussian Random Matrix (OGRM) based on polarized characteristics, and they classified anomalous behaviors using an enhanced Random Forest method [26].

However, machine learning-based methods for student classroom behavior recognition usually rely on manual feature extraction, which is a complex and time-consuming process of feature selection and extraction. Furthermore, there is a limited capacity to identify complicated and nonlinear behavioral patterns. Thus, in order to identify classroom behaviors, we shall employ deep learning-based techniques in this paper.

### 2.3 Recognizing classroom behavior using deep learning techniques

Computer vision-based student behavior recognition in the classroom has become a hotspot for study due to the rapid advancement of deep learning technologies. The two primary categories of mainstream approaches are object detection and human posture estimation.

Human posture estimation is the process of analyzing human posture and activity by using deep learning algorithms to determine the positions and angles of human joints in films or photographs. For instance, Jianwen Mo et al. employed a classification network to address the issue of identifying students’ classroom actions in classroom settings using a multi-task learning algorithm for object identification and human posture estimate tasks [27]. Zejie Wang et al. can identify and evaluate student behavior based on local features of interactive objects extracted by the YOLO v3 algorithm and global features of human posture extracted by the Openpose method. characteristics and student behavior can be found and examined to increase the accuracy of the recognition [6]. A system for recognizing student conduct based on person detection and skeletal position estimate was proposed by Feng-Cheng Lin et al. The OpenPose framework was utilized to gather skeletal data, and feature extraction was carried out to produce feature vectors that depict human postures. The suggested system was able to identify the number of pupils in the classroom and build a deep neural network to categorize the activities [2].

Nevertheless, the time it takes to identify information about the human skeleton somewhat slows down the detection speed. Secondly, in classroom scenarios, occlusion may result in inadequate detection of human skeleton information, which impacts the detector’s recognition accuracy [ 28]. Therefore, real-time performance and accurate recognition may be guaranteed by immediately learning features from photos through object detection. As an illustration, Haiwei Chen and colleagues presented an enhanced YOLOv8 classroom detection model. Firstly, a new module called C2f_Res2block is suggested. To improve detection performance, this module is incorporated with MHSA and EMA into the YOLOv8 model [12]. However, the baseline model’s simultaneous addition of several attention mechanisms results in a rise in model parameters and a fall in computational speed. In order to solve this issue, a few lightweight models have been developed with the goal of enhancing detection speed and computational efficiency while simultaneously improving the accuracy of student behavior recognition [29,30].

## 3. Materials and methods

### 3.1 Overview of IMRMB-Net

**Fig 2:**
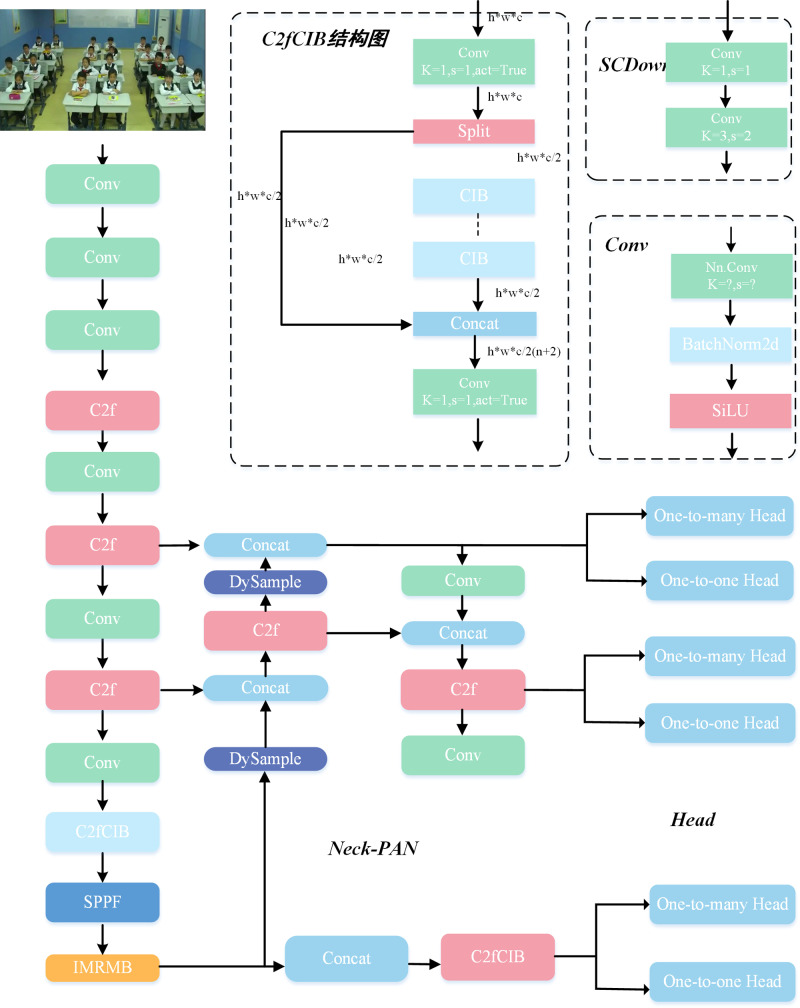
IMRMB-Net overall framework.

In this study, we propose an efficient and lightweight student behavior recognition model (IMRMB-Net) in complex classroom scenarios, aiming to solve the problems of occlusion, small objects, and interfering factors in classroom environments while improving the detection performance as well as decreasing the number of parameters and increasing the detection speed. The overall framework is shown in Fig 2.

Specifically, first, we design a lightweight module, IMRMB, that can increase processing speed while maintaining high accuracy by optimizing the feature extraction and recognition process. Second, we use the DySample dynamic upsampler in the neck network to make the model better for detection in classroom-dense scenarios. Finally, we design the Focaler-ShapeIoU loss function to enhance the focus on difficult-to-classify behaviors, better handle class imbalance in classroom behaviors, and improve the model’s detection accuracy and robustness to provide more accurate and reliable recognition results.

### 3.2 Invariant multi-level convolutional attention

In this study, based on Inverted Residual Mobile Block (iRMB), by combining Mixed Local Channel Attention (MLCA) to synthesize the channel information and spatial information, we propose a lightweight feature extraction Inverted Mix Residual Mobile Block (IMRMB).

#### 3.2.1 Inverted residual mobile block.

**Fig 3:**
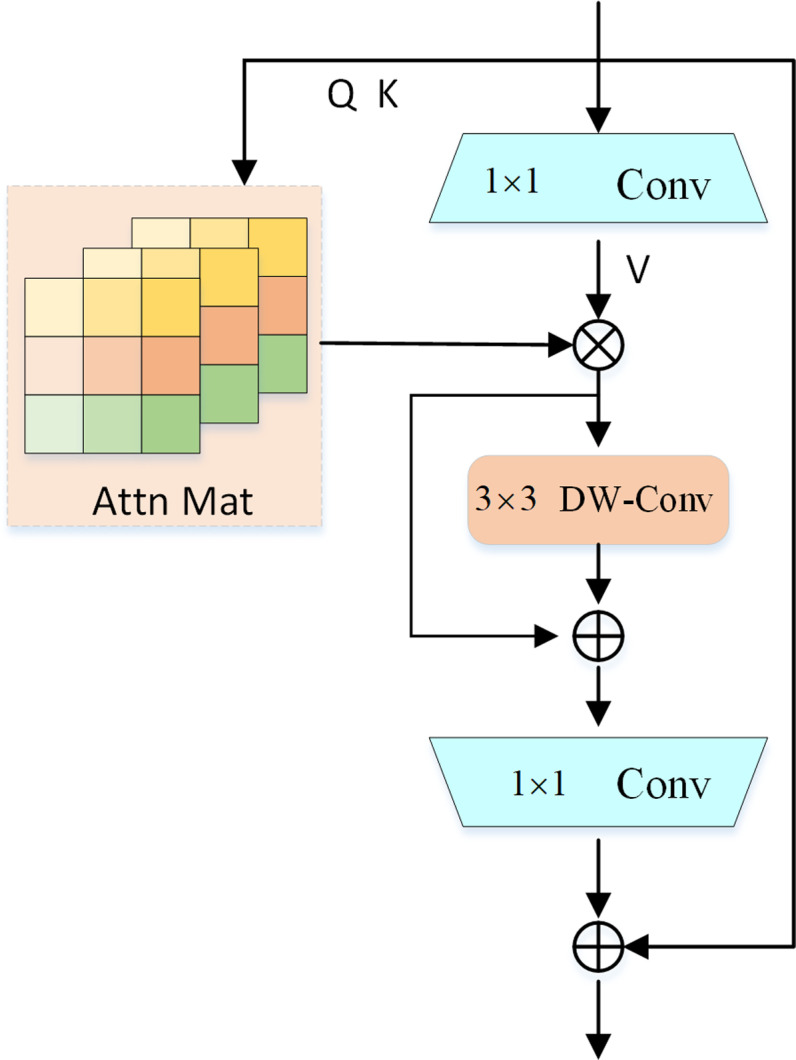
Inverted Residual Mobile Block structure diagram.

For dense prediction applications, the Inverted Residual Mobile Block (iRMB) structure combines the dynamic modeling capabilities of a Transformer with the lightweight nature of CNNs [31]. iRMB structure is shown in Fig 3.

The design objective of iRMB is to retain the model’s low weight while achieving high accuracy and effective use of computational resources. When extracting features, iRMB is able to catch the global relationships between various sections of the input data, whereas classic CNNs typically only capture local features. This allows iRMB to handle long-range information more successfully.

F in iRMB is modeled as a cascaded EW-MHSA and DW-Conv convolution:


$$F\left(\cdot \right)=\left(DW-Conv,Skip\right)\left(EW-MHSA\left(\cdot \right)\right)$$ (1)

Taking the image input $X\left(\in {\mathbb{R}}^{C\times H\times W}\right)$ as an example, MMB first extends the channel dimensions using an extension $MLP_{e}$ with an output/input ratio of *λ*:


$$X_{e}=MLP_{e}\left(X\right)\left(\in {\mathbb{R}}^{\lambda C\times H\times W}\right)$$ (2)

Then, intermediary operators like dynamic MHSA, static convolution, constant operator, etc. improve the picture features even more. Considering that MMB is suitable for efficient network design, we denote the concept of *F* as an efficient operator as:


$$X_{f}=F\left(X_{e}\right)\left(\in {\mathbb{R}}^{\lambda C\times H\times W}\right)$$ (3)

Finally, an inverse input/output ratio equal to *λ* shrinks $MLP_{s}$ to shrink the channel size:


$$X_{s}=MLP_{s}\left(X_{f}\right)\left(\in {\mathbb{R}}^{C\times H\times W}\right)$$ (4)

where the residual connection is used to obtain the final output:


$$Y=X+X_{s}\left(\in {\mathbb{R}}^{C\times H\times W}\right)$$ (5)

#### 3.2.2 Mixed local channel attention.

**Fig 4:**
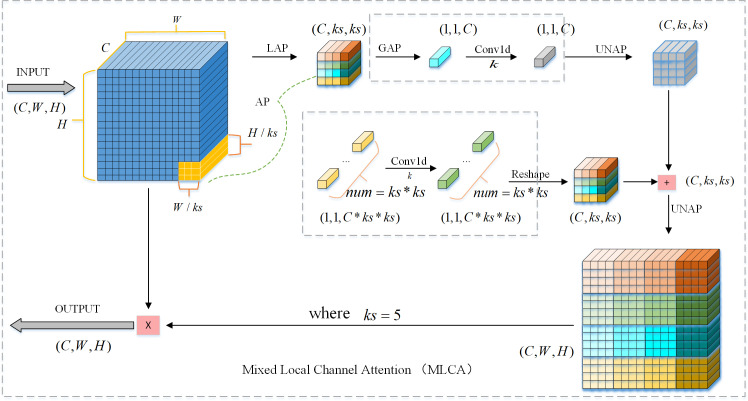
Structure diagram of Mixed Local Channel Attention.

One of the most popular parts of computer vision that aids neural networks in highlighting crucial information and squelching unimportant information is attention mechanisms. Spatial attention modules are typically complicated and expensive, and the great majority of channel attention methods only include channel feature information while ignoring spatial feature information. This results in subpar model representation outcomes or object recognition performance. A lightweight Mixed Local Channel Attention (MLCA) module can be used to improve the object detection network’s performance by striking a balance between complexity and performance. It can integrate local and global information, as well as channel and spatial information, to enhance the network’s representation [32]. The MLCA network structure is shown in Fig 4.

### 3.3 DySample

**Fig 5:**
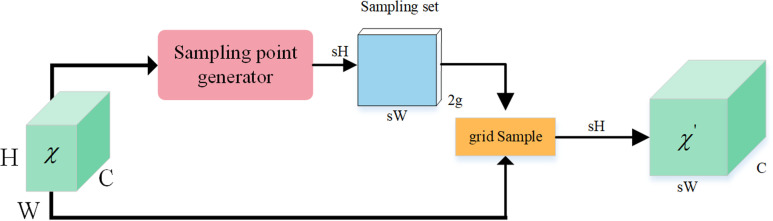
Sampling-based dynamic upsampling.

**Fig 6:**
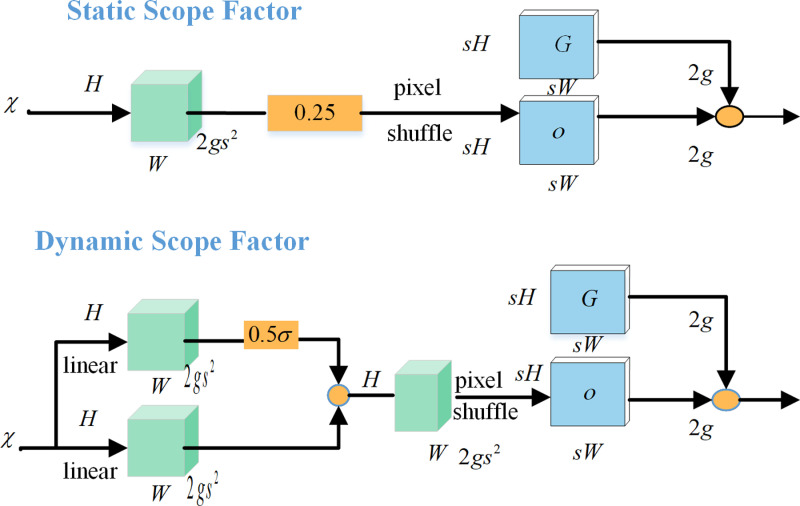
Sampling point generator in DySample.

The goal of DySample, a dynamic upsampler that is lightweight and effective, is to learn upsampling by sampling [33]. In contrast to conventional dynamic upsampling techniques that rely on convolutional kernels, DySample adopts a point sampling viewpoint, wherein a single point is divided into many points in order to attain more precise edges. The main technological idea is to use dynamic sampling to implement the upsampling procedure without requiring extra CUDA libraries. In order to do effective upsampling, DySample finds the appropriate semantic clustering for each upsampled point. DySample is tailored for up-sampling by sampling one point for each up-sampling site and dividing the points into $s^{2}$ up-sampling points, in contrast to techniques like UpSample. With a small increase in training time, DySample’s backpropagation is quick because of its highly optimized PyTorch built-in routines. DySample outperforms conventional dynamic upsamplers in a number of demanding prediction tasks, such as monocular depth estimation, semantic segmentation, object recognition, instance segmentation, and panoramic segmentation. The DySample structure is shown in Figs 5 and 6.

### 3.4 Focaler-ShapeIoU loss function

To address the problem of imbalance in student behavior samples in the UK_Dataset dataset, this study combines the Focaler-IoU loss function with the Shape-IoU loss function to form the Focaler-ShapeIoU as the loss function for identification.

#### 3.4.1 Focaler-IoU loss function.

In edge regression, the issue of uneven training samples persists. Depending on whether or not they contain the object category, the training samples can be divided into positive and negative categories. Focaler-IoU reduces the weight of samples that are easy to categorize, hence increasing the emphasis on samples that are harder to categorize. This is particularly crucial for recognizing student behavior in the classroom since Focaler-IoU can assist the model in more accurately identifying behaviors that are difficult to discriminate, such as those that have a high degree of similarity or ambiguity. There can be an imbalance in the number of samples from various behavioral categories when it comes to classroom behavior recognition. By assigning rare categories more weight, Focaler-IoU reduces the negative effects of category imbalance on model training and enhances the model’s capacity to identify behaviors from a small number of categories [34].

By reconstructing the IoU loss using a linear interval mapping technique, Focaler-IoU enhances edge regression by enabling it to concentrate on different regression samples for different detection tasks. The following is the formula:


$$IoU^{focaler}= \{\begin{array}{l} 0,Iou<d\\ \frac{\left(IoU-d\right)}{u-d}\\ 1,IoU>u \end{array},d\leq IoU\leq u$$ (6)

where $IoU^{focaler}$ is the Focaler-IoU after reconstruction, IoU is the original IoU value, and [d,u]∈[0,1]. Adjusting the values of *d* and *u* can make $IoU^{focaler}$ focus different regression samples. The loss is defined as follows:


$$L_{Focaler-IoU}=1-IoU^{focaler}$$ (7)

Using Focaler-IoU with the current edge regression loss methods based on IoU:


$$L_{Focaler-GIoU}=L_{GIoU}+IoU-IoU^{Focaler}$$ (8)

$$L_{Focaler-DIoU}=L_{DIoU}+IoU-IoU^{Focaler}$$ (9)

$$L_{Focaler-CIoU}=L_{CIoU}+IoU-IoU^{Focaler}$$ (10)

$$L_{Focaler-EIoU}=L_{EIoU}+IoU-IoU^{Focaler}$$ (11)

$$L_{Focaler-SIoU}=L_{SIoU}+IoU-IoU^{Focaler}$$ (12)

#### 3.4.2 Shape-IoU loss function.

Shape-IoU measures how closely the predicted results match the actual label shapes. The model can more precisely locate and identify student behaviors by optimizing the Shape-IoU Loss, which raises the recognition accuracy overall [35]. The bounding box regression loss is as follows:


$$L_{Shape-IoU}=1-IoU+dis\tan ce^{shape}+0.5\times \Omega^{shape}$$ (13)

$$dis\tan ce^{shape}=hh\times {\left(x_{c}-x_{c}^{gt}\right)}^{2}/c^{2}+ww\times {\left(y_{c}-y_{c}^{gt}\right)}^{2}/c^{2}$$ (14)

$$\Omega^{shape}={\sum_{t=w,h}\left(1-e^{-wt}\right)}^{\theta },\theta =4$$ (15)

$$ww=\frac{2\times {\left(w^{gt}\right)}^{scale}}{{\left(w^{gt}\right)}^{scale}+{\left(h^{gt}\right)}^{scale}}$$ (16)

$$hh=\frac{2\times {\left(h^{gt}\right)}^{scale}}{{\left(w^{gt}\right)}^{scale}+{\left(h^{gt}\right)}^{scale}}$$ (17)

where scale is the scaling factor, which is related to the size of the object in the dataset, and ww and hh denote the weight coefficients in the horizontal and vertical directions, respectively, and their values are related to the shape of the GT box.

In conclusion, the Focaler-ShapeIoU loss function, which is created by combining Focaler-IoU and Shape-IoU, can simultaneously account for the difficulty of behavior classification and shape similarity, allowing the model to be optimized for both localization and classification and improving overall performance. Furthermore, the accuracy of behavior recognition is impacted by light, angle, and occlusion in actual classroom settings.Focaler-ShapeIoU optimizes shape similarity and focuses on hard-to-classify samples to make the model perform more reliably in the face of these perturbing effects.

## 4. Experiments

This section describes the dataset, experimental setup, and experimental results. Next, we evaluate our method against a number of popular object detection systems. Ultimately, an array of ablation tests is carried out to confirm the respective contributions of the constituents inside the suggested IMRMB-Net.

### 4.1 Experimental setup and datasets

#### 4.1.1 Experimental settings.

The experiments were carried out on a server running Ubuntu 22.04 with an NVIDIA GeForce GTX 3090 GPU, an Intel(R) Xeon(R) Gold 6152 CPU * 10-core CPU, and an NVIDIA GPU. Python 3.10.14 and the Pytorch2.0.1 deep learning framework are used in this investigation. Furthermore, we employed CUDA 12.1. The experiments reported in this work were all designed with an early stop mechanism that caused training to end early if average accuracy did not increase considerably after 5 epochs. The studies were intended to train for 200 epochs. By monitoring the validation set error during training and stopping training early when the validation error starts to climb, the early stopping mechanism is a useful tactic to prevent the overfitting of deep learning models [36]. Furthermore, the batch size of 32, the learning rate of 0.01 during model training, the optimizer of SGD, the SGD momentum of 0.937, and the optimizer weight decay of 0.0005 were all set throughout the model’s training in this study.

#### 4.1.2 Description of the datasets.

**Fig 7:**
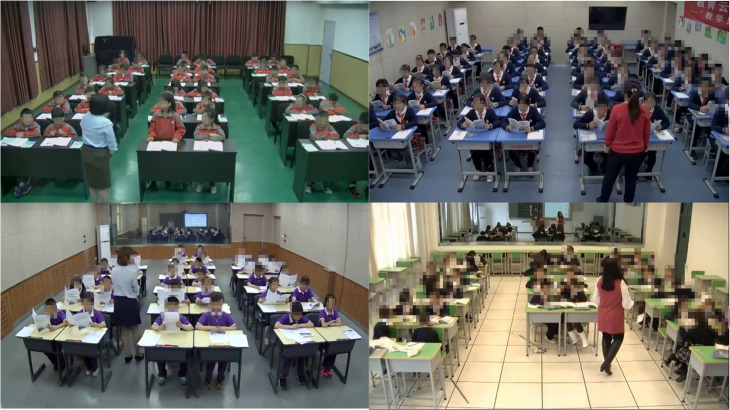
Map of occlusion present in real classroom scenarios.

This study verifies the validity of IMRMB-Net by conducting experiments on a self-constructed classroom behavior dataset (UK_Dataset) and a publicly available dataset SCB_Dataset [37].UK_Dataset is derived from the 2019 elementary school classroom videos collected from the National Education Resources Public Service Platform (NERPSP), and the classroom scenes in the videos have a large number of student-teacher occlusion, mutual occlusion between students and mutual occlusion between students and objects in the classroom, as shown in Fig 7. We intercepted 8754 images by frames, and considering the detection needs in real classroom scenarios, we classified these images into eight categories of typical student behaviors: writing, reading, listening, raising hands, turning, standing, discussing, and accepting instructions from the teacher. The criteria for defining the eight behaviors are outlined in Table 1. SCB_Dataset, a student classroom behavior dataset proposed by Yang[38], contains multiple shooting angles and most of the images are dense and occluded from each other. We divide the UK_Dataset and SCB_Dataset datasets into training, validation, and testing sets in the ratio of 7:2:1.

**Table 1:** Categories and descriptions of student behavior.

Category	Description
Discuss	Some of the students converse with one another.
Standing	Students respond to questions while standing.
Raise_hand	Students raise their hands, left or right.
Listen_to_lectures	Students pay attention to the teacher’s talk.
Read_book	Students lowered their heads and read the textbook.
Turned	Students rotate their bodies and talk to their classmates.
Take_notes	Students lower their heads to write.
Teacher_explain	The teacher guides the students beside them.

**Fig 8:**
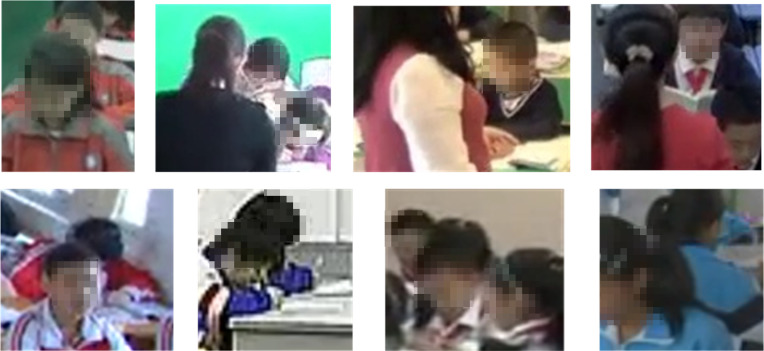
Typical occlusion scene.

To further investigate the ability of IMRMB-Net to solve occlusion problems in classroom scenarios, we categorized the test set portions of UK_Dataset and SCB_Dataset into two categories according to the degree of occlusion: “Heavy Occlusion (HO)” (visibility less than or equal to 75%) and “Low Occlusion (LO)” (visibility greater than 75%). Fig 8 shows a typical occlusion scenario, from which it can be observed that the student’s face and upper body are often difficult to recognize clearly due to occlusion or distance. With the above quantitative metrics of occlusion and subject differentiation, we further experimentally tested the model’s performance in groups, comparing the model’s accuracy changes in high and low occlusion scenarios, thus verifying the model’s adaptability and advantages in dealing with occlusion scenarios.

### 4.2 Evaluation indicators

This study uses Precision (mAP), number of parameters, GFLOPs, and FPS to evaluate model performance.


$$Precision=\frac{TP}{TP+FP}$$ (18)

$$Recall=\frac{TP}{TP+FN}$$ (19)

$$AP=\int_{0}^{n}\left(Precision\right)d\left(Recall\right)$$ (20)

$$mAP=\frac{\sum_{0}^{j}AP}{j}$$ (21)

where *n* and *j* denote the total number of instances of all classes and specific classes, respectively.

To evaluate the accuracy of the model, the mAP evaluation metric is used, where mAP50 is the mAP calculated at 50% of the IoU threshold, which is more suitable for quickly evaluating the basic performance of the model. mAP@50:95, on the other hand, indicates the average accuracy calculated at multiple IoU thresholds (from 50% to 95%, with a step of 5% each time), which is a more stringent evaluation criterion and can more truly reflect the model’s performance in complex scenarios[39].

Furthermore, it is imperative to take into account the detection speed, parameter sizes, and GFLOPs while assessing the lightweight qualities of the detection model [4]. For lightweight models, the number of parameters is very important, and the magnitude of the number of parameters directly affects the amount of computing needed for model inference and training. Reducing the number of model parameters can increase computational efficiency, lower resource consumption, and speed up the model’s completion of training and inference tasks on devices with limited resources. Thus, model lightweight can be obtained throughout the model lightweight acceleration process while keeping model performance intact by judiciously lowering the number of parameters. The computational complexity of an algorithm can be measured using GFLOPs, or global factor of performance. Each model’s ability to identify objects in real-time is measured in frames per second or FPS. The number of frames that the model can handle in a second is known as the frame rate per second (FPS), and this parameter measures how quickly the model operates. The model can be used on hardware with relatively modest processing capacity because a higher FPS indicates that the model is less computationally demanding [40]. Using a batch size of one for each measurement, the average of five FPS readings is computed in this study.

### 4.3 Experimental results

#### 4.3.1 Experimental results for the UK_Dataset dataset.

In this study, IMRMB-Net was firstly compared and analyzed with five widely used target detection methods on the UK_Dataset dataset, including YOLOv5, YOLOv8[41], YOLOv7-tiny, Faster-RCNN, and YOLOv10s [42], to validate that IMRMB-Net has a certain degree of model complexity aspect and performance advantages. The comparison results on the UK_Dataset are shown in Table 2. The best results for each dataset are highlighted in bold.

**Table 2:** Comparison results on UK_Dataset.

Models	mAP@50(%)	mAP@50:95(%)	FPS	GFLOPs(G)	Params(MB)
Faster-RCNN	91.2	74.8	54.60	78.7	60.74
YOLOv7-tiny	92.0	72.5	96.56	13.2	6.03
YOLOv8s	91.8	76.3	69.78	28.5	11.10
YOLOv10s	92.0	76.1	58.24	24.5	8.04
YOLOv5s	91.5	76.2	62.62	23.9	9.11
ours	93.3	78.7	60.37	23.8	7.32

The experimental results show that the recognition accuracy of IMRMB-Net is better than the other compared models, and IMRMB-Net achieves 93.3% and 78.7% in mAP@50 and mAP@50:95 evaluation metrics, which is 1.3% higher than YOLOv10 model. In addition, it is 1.3% higher than YOLOv7-tiny, 1.5% higher than YOLOv8s, 2.1% higher than Faster-RCNN, and 1.8% higher than YOLOv5s. Mutual occlusion amongst students is a major issue in classroom settings generally, and pupils seated at the back corners of the room are harder to identify because of their small pixel sizes and less noticeable visual characteristics. Specifically, IMRMB is introduced to enhance the accuracy of occluded object recognition by capturing contextual information while taking channel and spatial information into account. With the addition of Dysample, IMRMB-Net is able to recognize small objects more accurately than any of the other approaches that were examined.

In addition, in order to evaluate the lightweight properties of the detection model, the detection speed, parameter sizes, and GFLOPs should also be considered. In this study, IMRMB is introduced, which combines the information of local and global features, as well as channel and spatial features, and is able to increase only a small number of parameter sizes and GFLOPs with a substantial improvement in the detection accuracy. Dynamic convolution is bypassed by utilizing DySample, and up-sampling is expressed in terms of point samples. perspective to represent upsampling, saving computational resources. The experimental results show that the number of parameters of IMRMB-Net is 7.32 MB, which is only 1.29 MB higher than the first lightweight model YOLOv7-tiny (6.03 MB), 53.42 MB smaller than Faster-RCNN (60.74 MB), 3.78 MB smaller than YOLOv8s (11.1 MB), and 3.78 MB smaller than YOLOv10s (8.04MB), 0.72MB smaller than YOLOv5s (9.11MB), and 1.79MB smaller than YOLOv5s (9.11MB). IMRMB-Net’s GLOPs (23.8) ranked the second largest, 10.6 larger than the top-ranked YOLOv7-tiny (13.2), 54.9 smaller than Faster-RCNN (78.73), and 4.5 smaller than YOLOv8s (28.5), 4.7 smaller than YOLOv10s (24.5), 0.7 smaller than YOLOv10s (24.5), and 0.1 smaller than YOLOv5s (23.9). The above results indicate that IMRMB-Net has a smaller model size and computational complexity, which enables it to perform the training and inference tasks more optimally on resource-constrained devices such as cameras.

#### 4.3.2 Validation of the masking problem.

In order to further validate the effectiveness of IMRMB-Net in solving the occlusion problem in classroom scenarios, we validate the occlusion subsets divided by IMRMB-Net on UK_Dataset and SCB_Dataset in comparison with the baseline model, which includes UK_HO, UK_LO, SCB_HO, and UK_LO. The comparison results are in Table 3.

**Table 3:** Comparison of the masked subsets divided on UK_Dataset and SCB_Dataset with the baseline model, the masked subsets include UK_HO, UK_LO, SCB_HO, and UK_LO.

Models	UK_LO: mAP@50(%)	UK_HO: mAP@50(%)	SCB_LO: mAP@50(%)	SCB_HO: mAP@50(%)
YOLOv10s(baseline)	93.1	87.8	81.4	70.2
**IMRMB-Net**	**93.7**	**89.1**	**82.6**	**72.5**

The experimental results show that the mAP@50 of IMRMB-Net in the high occlusion subset of UK_Dataset is improved by 1.3% compared to the baseline model, which is much larger than the 0.6% improvement in the low occlusion subset, and the improvement of the mAP@50 of the high occlusion subset of SCB_Dataset is 2.3%, and the improvement of the low occlusion is 1.2%, which are two sets of experiments that show that the IMRMB-Net proposed in this study model has better effect enhancement in the high occlusion case.

**Fig 9:**
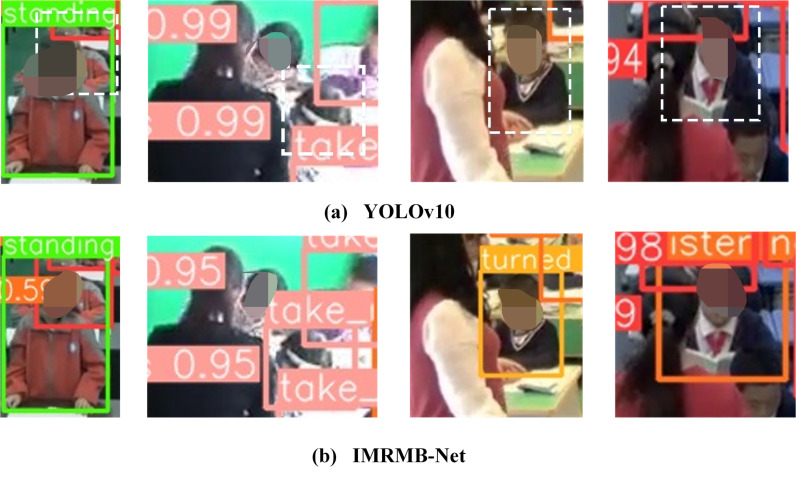
IMRMB-Net detection effect graph for UK_Dataset with high occlusion factor.

**Fig 10:**
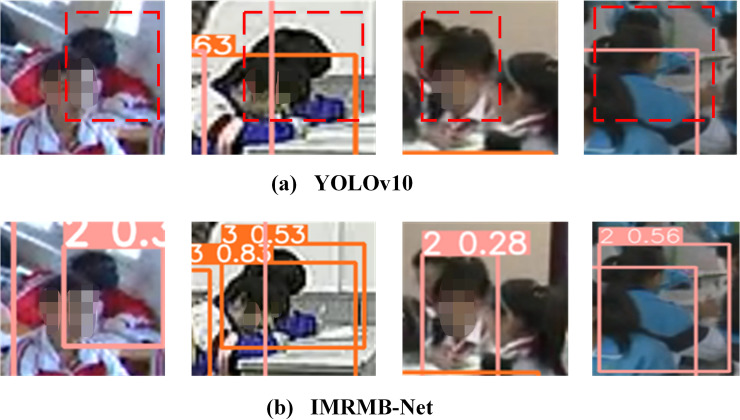
IMRMB-Net detection effect graph for SCB_Dataset with high occlusion factor.

It is verified that IMRMB-Net has a good effect in dealing with high occlusion problems, the comparison detection graph of high occlusion is shown in Figs 9 and 10. Meanwhile, the accuracy of UK_LO and SCB_LO models in low occlusion is also improved, which shows that for low occlusion IMRMB-Net can also be effective in detecting the case.

#### 4.3.3 Validation of the small target problem.

**Fig 11:**
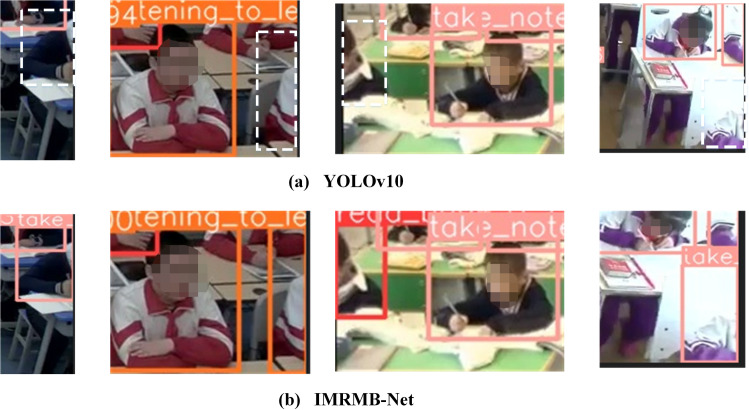
IMRMB-Net’s small target detection effect graph in the test set.

The problem of small target detection is another big problem of student behavior detection, UK_Dataset contains some of the small targets of students in the corners, we analyze the detection effect graphs of IMRMB-Net in the test set, and conclude that IMRMB-Net can solve the problem of small target detection to a certain extent, and the detection effect is shown in Fig 11.

In order to evaluate the ability of IMRMB to recognize small objects and its generalization ability, we introduce VisDrone2021[43].The VisDrone2021 dataset contains a large number of small objects such as pedestrians, vehicles, bicycles, etc. The experimental results on VisDrone2021 are shown in Table 4.

**Table 4:** Comparison Results of VisDrone2021 Dataset.

Models	mAP@50(%).	mAP@50:95(%)	FPS	GFLOPs(G)	Params(MB)
YOLOv10s(baseline)	36.0	21.2	50.44	24.5	8.04
IMRMB-Net	38.0	22.7	52.65	23.9	7.32

The mAP@50 and mAP@50:95 of IMRMB-Net on VisDrone2021 were 38.0% and 22.7%, respectively, which were both higher than the baseline model YOLOv10s (mAP@50=36.0%, mAP@50:95=21.2%). Among them, mAP@50 is 2.0% higher and mAP@50:95 is 1.5% higher. In addition, IMRMB-Net also outperforms the baseline model in terms of lightweight performance. Among them, FPS is 2.21 higher, GFLOPs is 0.6G lower, and Params is 0.72MB lower.

**Fig 12:**
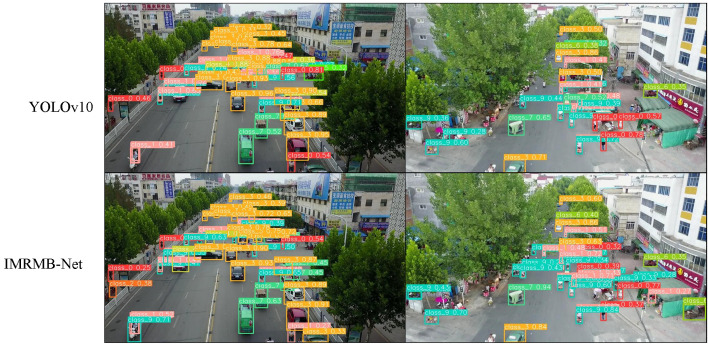
Visual comparison of YOLOv10 and IMRMB-Net on the VisDrone2021 dataset.

Fig 12 displays a comparison of the VisDrone2021 dataset’s recognition visualizations using YOLOv10 and IMRMB-Net. Apart from having a better recognition accuracy, IMRMB-Net can identify a greater number of small objects in the UAV photos.

Drawing from the aforementioned experimental findings, it can be inferred that IMRMB-Net is a highly effective solution for identifying classroom behaviors in addition to being a good object detector for datasets with a large number of small objects, like VisDrone 2021. Furthermore, the outcomes demonstrate the outstanding generalization potential of IMRMB-Net.

### 4.4 Ablation study

In order to fully validate the effectiveness of the IMRMB-Net model proposed in this study, we conducted detailed ablation experiments on the UK_Dataset dataset.

#### 4.4.1 Impact of the IMRMB module.

To evaluate the effectiveness of the proposed IMRMB module, we implant the IMRMB module in the backbone network of the baseline model. In this section, we not only investigate the impact of the IMRMB module on the complexity and performance aspects of the baseline model but also explore the effectiveness of IMRMB in addressing mutual occlusion. The experimental results in terms of model complexity and overall performance are shown in Table 5. The IMRMB structure designed in this study improves the recognition accuracy and reduces the computational resources. Among them, mAP@50 increased from 92.0% to 92.9%, mAP@50:95 is improved from 76.1% to 78.2%, FPS is improved from 58.24 to 61.69, GFLOPs is reduced from 24.5(G) to 23.9(G), Params is reduced from 8.04(MB) to 7.32(MB). For the experimental results of resolving the occlusion effect, as shown in Table 6. the baseline model after implanting the IMRMB module improves by 0.1% in mAP@50 for the experimental case of low occlusion UK_LO and improves by 0.8% for the experimental case mAP@50 of high occlusion UK_HO.

**Table 5:** Comparative results of ablation experiments on the UK_Dataset.

Model	mAP@50(%)	mAP@50:95(%)	FPS	GFLOPs(G)	Params(MB)
YOLOv10s(Baseline)	92.0	76.1	58.24	24.5	8.04
+IMRMB	92.9(↑0.9)	78.2(↑2.1)	61.69(↑3.45)	23.9(↓0.6)	7.32(↓0.72)
+DySample	92.1(↑0.1)	76.4(↑0.3)	59.12(↑0.88)	24.5	8.04
+Focaler-ShapeIoU	92.9(↑0.9)	77.9(↑1.8)	60.28(↑2.04)	24.5	8.04
+IMRMB+DySample	93.2(↑1.2)	78.0(↑1.9)	59.26(↑1.02)	23.9(↓0.6)	7.32(↓0.72)
+IMRMB+DySample+Focaler-ShapeIoU**(Ours)**	93.3	78.7	60.37	23.9	7.32

**Table 6:** An ablation experiment to solve the occlusion problem.

Model	UK_LO:mAP@50(%)	UK_HO:mAP@50(%)
YOLOv10s(Baseline)	93.1	87.8
+IMRMB	93.2	88.6
+IMRMB+DySample	93.4	88.6
**Ours (**+IMRMB+DySample+Focaler-ShapeIoU**)**	93.7	89.1

#### 4.4.2 Impact of the dysample module.

In this subsection, we first validate the effectiveness of the IMRMB module by further introducing the DySample module on top of the implanted IMRMB module. The results in terms of model complexity and overall performance are shown in Table 5. mAP@50 improves the baseline model from 92.9% to 93.2% over the implanted IMRMB module. Meanwhile, we conducted experiments in UK_HO and UK_LO data subsets, and the experimental results proved that, although there is no obvious improvement in UK_HO, there is a 0.2% improvement in UK_LO compared with the implanted IMRMB, which is due to the fact that DySample can enable the network to focus on the target area more flexibly by dynamically adjusting the sampling position, which is more effective for the detection of the dense away scenario.

#### 4.4.3 Impact of the Focaler-ShapeIoU module.

Based on the system of introducing IMRMB and DySample, we finally introduce the Focaler-ShapeIoU, and the results show that the final introduced Focaler-ShapeIoU mAP@50 improves from 93.2% to 93.4%, mAP@50 improves from 78.0% to 78.7%, and the FPS improves from 59.26 to 60.37. In the case of UK_ HO and UK_LO data subsets the experiments proved that the final introduced Focaler-ShapeIoU has a good effect in solving the occlusion problem, and for the baseline model with the introduction of IMRMB and DySample, it improves by 0.3% in the UK_LO data subset and by 0.5% in the UK_HO data subset.

## 5. Conclusion

Student behavior recognition in complex classroom scenarios faces many challenges, including occlusion, small objects, category imbalance, distractors, and limited resources. To address these challenges, this study proposes a lightweight student behavior recognition model (IMRMB-Net) for complex classroom scenarios. In this study, a new lightweight attention mechanism IMRMB is proposed by combining iRMB with MLCA. IMRMB can help the detection model better capture the contextual information, solve the problem of students’ occlusion of each other in the classroom scenario, and effectively improve the performance and speed of the model detection. In addition, this study uses the DySample structure in the neck network to realize dynamic sampling by point sampling, which improves the detection ability of small objects in the course scene. In this study, Focaler-IoU and Shape-IoU are combined to form the Focaler-ShapeIoU loss function, which makes the model more effective in facing classroom disturbances including occlusion by focusing on hard-to-classify samples and optimizing shape similarity.

In this study, IMRMB-Net shows a large advantage in recognition accuracy and lightweight performance (number of parameters, FPS, GFLOPs) by comparing UK_Dataset with the other five models. By conducting experiments on the self-constructed classroom behavior dataset UK_Dataset and SCB_Dataset dataset divided into occlusion subsets, it is verified that IMRMB-Net can effectively solve the occlusion problem in classroom scenarios. In addition, this study also verifies the ability and generalization of IMRMB-Net to recognize small targets on a publicly available UAV image dataset (VisDrone2021).

In conclusion, IMRMB-Net offers a viable way to effectively and precisely identify objects in challenging classroom situations, thus enhancing both instructional research and classroom management. Future research will assess the IMRMB-Net model’s performance on increasingly complicated datasets.
